# Supplementary material for: A Probabilistic Structural Equation Model to Evaluate Links between Gut Microbiota and Body Weights of Chicken Fed or Not Fed Insect Larvae
Source: Biology (Basel). 2022 Feb 23;11(3):357. doi: 10.3390/biology11030357 (PMC8945536; doi:10.3390/biology11030357)
Supplement: Supplementary file 1 [file biology-11-00357-s001.zip › biology-1592735-supplementary.pdf]

Table S1. Ingredients (%) and chemical (g/kg DM) of the control experimental diets.

	Control	BSF5	BSF10
Ingredients (%)			
Corn	30.00	30.50	31.00
Maize	30.00	31.92	33.75
Rebulet	4.00	4.50	5.00
Pois	4.00	4.00	4.00
Soybean meal	20.20	13.50	7.00
Sunflower meal	3.00	3.00	3.00
Rapeseed meal	2.00	2.00	2.00
Chalk	2.00	2.00	2.00
Salt	0.20	0.20	0.10
Monocalcim Phosphate	0.40	0.25	0.10
Oil	2.50	1.45	0.40
L-Methionine	0.20	0.18	0.15
Mineral and vitamin complex	1.50	1.50	1.50
BSF larvae	0.00	5.00	10.00
Chemical composition			
Humidity	12.36	12.65	12.95
Ash	7.86	7.53	7.20
Ethereal extract	7.79	7.54	7.30
Crude cellulose	5.05	5.15	5.25
Lysine	0.86	0.91	0.95
Methionine	0.46	0.48	0.50
Crude protein	17.37	17.56	17.75
Metabolizable energy (kcal/kg)	2822.61	2858.63	2894.65
Calcium	0.96	0.96	0.97

Phosphore	0.51	0.53	0.55

Table S2. Mean relative abundances (%) of each OTU genus per sex (Female or Male) and age (in days) of the birds, per origin of the sample (Caecum (C) or cloaca (L)), and per percent of *Hermetia Illucens* (BSF) in the diet (0%, 5% or 10%).

**Origin of the sample**	**C**	**C**	**C**	**C**	**C**	**C**	**C**	**C**	**C**	**C**	**C**	**C**	**C**	**C**	**C**	**C**	**C**	**C**	**L**	**L**	**L**	**L**
**Sex**	**F**	**F**	**F**	**F**	**F**	**F**	**F**	**F**	**F**	**M**	**M**	**M**	**M**	**M**	**M**	**M**	**M**	**M**	**F**	**F**	**M**	**M**
**Age (days)**	**39**	**39**	**39**	**67**	**67**	**67**	**73**	**73**	**73**	**39**	**39**	**39**	**67**	**67**	**67**	**73**	**73**	**73**	**16**	**28**	**16**	**28**
**BSF (%)**	**0**	**5**	**10**	**0**	**5**	**10**	**0**	**5**	**10**	**0**	**5**	**10**	**0**	**5**	**10**	**0**	**5**	**10**	**0**	**0**	**0**	**0**
Corynebacterium 1	0.03	0.03	0.11	0.00	0.00	0.00	0.01	0.00	0.00	0.10	0.07	0.04	0.00	0.00	0.00	0.00	0.00	0.02	0.01	0.25	0.00	1.17
Bacteria	0.05	0.25	0.03	1.15	1.38	1.05	0.08	0.31	0.16	0.01	1.58	0.31	1.40	1.54	0.97	0.11	0.13	0.30	0.02	0.06	0.30	0.01
Bacteroides	0.00	0.10	0.20	0.29	1.93	45.54	0.76	0.39	0.50	0.00	0.19	0.26	2.27	0.91	39.77	0.60	1.04	0.39	0.01	0.03	0.01	0.03
Bacteroidales	0.00	0.00	0.00	0.04	0.12	0.99	0.00	0.00	0.01	0.00	0.00	0.00	0.09	0.12	0.74	0.00	0.01	0.00	0.00	0.00	0.00	0.00
Barnesiella	0.00	0.00	0.00	0.13	1.05	2.37	0.00	0.00	0.00	0.00	0.00	0.00	0.15	1.42	1.85	0.00	0.00	0.00	0.00	0.00	0.01	0.00
Alistipes	0.00	0.00	0.01	23.76	43.02	16.97	1.83	0.83	0.95	0.00	0.01	0.02	22.93	35.60	14.83	3.66	3.03	1.31	0.01	0.00	0.00	0.47
Gastranaerophilales	1.13	2.03	0.44	7.19	1.08	0.06	0.68	0.94	0.61	0.11	2.02	1.58	3.05	0.39	0.81	0.33	0.69	0.88	0.00	0.19	0.01	0.07
Helicobacter	0.00	0.00	0.00	0.64	0.81	0.51	0.00	0.00	0.00	0.00	0.00	0.00	1.28	1.41	0.90	0.00	0.00	0.00	0.00	0.09	0.00	0.01
Enterococcus	0.08	0.05	0.09	0.00	0.00	0.00	0.01	0.00	0.07	0.09	0.13	0.08	0.00	0.03	0.02	0.00	0.00	0.09	1.05	0.19	0.09	4.59
Lactobacillus	44.51	24.34	76.56	0.07	0.10	0.29	34.40	0.72	24.64	44.82	44.94	51.91	0.16	0.44	0.13	0.84	2.06	20.89	51.52	83.38	51.95	62.64
Christensenellaceae R-7 group	0.05	0.32	0.23	0.54	0.53	0.20	0.37	0.44	0.64	0.04	0.31	0.33	0.30	0.54	1.05	0.80	1.00	0.81	0.02	0.01	0.01	0.00
Candidatus Arthromitus	3.29	4.45	0.73	0.01	0.02	0.00	3.71	0.04	1.38	19.05	4.01	0.68	0.02	0.03	0.00	0.06	0.02	0.76	7.20	3.85	15.93	11.39
Clostridiales	0.21	1.01	0.05	3.08	1.25	1.05	1.53	1.25	1.28	0.66	0.07	0.12	3.48	2.76	2.37	2.22	1.69	1.38	0.04	0.43	0.04	0.00
Clostridiales vadinBB60 group	0.13	0.81	0.41	3.13	1.05	0.35	1.90	2.57	2.64	0.25	2.19	1.40	0.72	1.16	1.81	2.48	4.08	3.39	0.30	0.08	0.36	0.13
Eisenbergiella	0.18	1.20	0.22	1.42	0.76	0.63	3.33	5.62	3.37	0.16	0.78	1.16	1.57	0.85	0.55	4.01	5.39	1.73	0.27	0.62	1.22	0.15
GCA-900066575	0.02	0.27	0.06	0.36	0.33	0.13	0.27	0.82	0.29	0.06	0.26	0.24	0.48	0.31	0.19	0.46	0.30	0.29	0.15	0.08	0.29	0.00
Lachnospiraceae	1.60	9.31	2.90	13.59	11.96	6.86	13.91	17.45	15.87	3.63	7.09	4.60	17.17	14.56	8.32	25.15	17.54	16.81	7.87	2.91	5.78	3.86
Sellimonas	0.05	0.40	0.06	0.25	0.26	0.12	0.16	0.33	0.19	0.04	0.09	0.16	0.24	0.22	0.15	0.46	0.23	0.21	0.12	0.06	0.15	0.25
Tyzzerella	0.02	0.31	0.10	0.17	0.27	0.16	0.12	0.37	0.23	0.04	0.19	0.44	0.47	0.30	0.13	0.52	0.62	0.26	0.11	0.02	0.10	0.00
Butyricicoccus	0.04	0.33	0.04	0.64	1.21	0.41	0.34	1.13	0.46	0.04	0.10	0.18	1.02	0.52	0.37	0.50	0.49	0.61	0.46	0.22	0.85	0.15
DTU089	0.20	0.76	0.58	0.13	0.08	0.09	0.23	0.58	0.27	0.32	0.49	0.55	0.23	0.11	0.07	0.20	0.29	0.20	0.28	0.03	0.79	0.06
Faecalibacterium	2.31	10.35	3.20	2.78	3.37	4.02	8.63	7.55	5.34	8.80	4.32	9.38	4.42	5.41	2.68	10.88	7.57	2.92	0.09	0.71	0.03	1.97
Flavonifractor	0.04	0.25	0.16	0.12	0.17	0.08	0.18	0.59	0.32	0.04	0.43	0.18	0.40	0.25	0.06	0.27	0.34	0.23	0.10	0.09	0.23	0.00

Fournierella	0.00	0.07	0.02	0.23	0.36	0.36	0.10	0.18	0.05	0.00	0.00	0.07	0.11	0.31	0.17	0.06	0.03	0.02	0.00	0.00	0.00	0.20
Negativibacillus	0.05	0.21	0.12	0.36	0.48	0.36	0.62	1.74	0.89	0.29	0.46	0.37	0.30	0.67	0.26	1.94	1.28	1.13	0.45	0.10	0.26	0.00
Oscillibacter	0.04	0.18	0.05	0.45	0.34	0.44	0.51	0.72	0.78	0.08	0.14	0.16	0.69	0.52	0.33	1.02	1.08	0.63	0.22	0.07	0.14	0.18
Ruminiclostridium 5	0.07	0.42	0.34	0.56	0.98	0.43	0.65	1.93	1.47	0.13	0.62	0.44	0.69	0.38	0.43	1.55	1.07	1.25	0.77	0.09	0.96	0.35
Ruminiclostridium 9	0.05	0.36	0.21	1.47	1.42	0.68	1.87	3.16	1.53	0.08	0.52	0.47	1.60	1.11	0.69	2.64	3.60	1.87	0.64	0.14	1.32	0.19
Ruminococcaceae	0.67	5.93	1.74	20.19	10.48	5.34	8.38	14.37	11.19	0.56	2.94	3.65	14.28	11.22	8.67	12.69	16.43	8.70	2.33	1.91	3.04	1.29
Ruminococcaceae NK4A214 group	0.04	0.09	0.22	0.74	0.72	0.50	0.58	1.21	1.06	0.03	0.42	0.21	0.41	0.82	0.66	0.62	1.12	0.75	0.00	0.00	0.01	0.00
Ruminococcaceae UCG-005	1.77	7.82	1.96	6.32	2.75	0.84	3.86	4.99	4.83	0.34	5.53	3.34	1.75	1.65	0.59	6.14	5.48	4.66	1.76	0.52	0.16	0.29
RuminococcaceaeCG-010	0.03	0.17	0.05	0.44	0.46	0.14	0.16	0.40	0.25	0.01	0.23	0.20	0.24	0.23	0.22	0.32	0.21	0.45	0.00	0.00	0.00	0.00
Ruminococcaceae UCG-014	0.65	1.74	1.61	5.12	3.68	4.32	3.37	13.25	10.00	0.14	3.71	2.27	6.95	5.20	5.65	9.81	9.38	6.84	2.20	1.17	2.24	0.79
Subdoligranulum	0.10	0.36	0.29	0.58	1.11	0.47	0.75	5.40	0.81	0.18	0.16	0.29	1.50	1.94	0.84	1.23	2.11	2.27	0.89	0.08	2.92	0.44
Erysipelatoclostridium	0.15	0.33	0.17	0.02	0.16	0.14	0.29	0.59	0.56	0.13	0.26	0.15	0.13	0.17	0.08	0.35	0.63	1.02	0.99	0.08	1.39	0.23
Megamonas	0.00	0.00	0.00	0.01	2.99	1.53	0.00	0.00	0.00	0.00	0.00	0.00	6.97	4.48	1.62	0.00	0.00	0.02	0.00	0.00	0.00	0.00
Candidatus Saccharimonas	0.00	0.09	0.04	1.34	0.26	0.12	0.00	0.00	0.00	0.04	0.10	0.05	0.11	0.07	0.09	0.00	0.00	0.00	0.00	0.00	0.00	0.00
Alcaligenes	0.02	0.00	0.00	0.00	0.00	0.00	0.24	0.01	0.26	0.10	0.00	0.01	0.00	0.00	0.00	0.01	0.01	3.86	10.42	0.10	1.05	2.59
Escherichia-Shigella	0.02	0.08	0.03	0.00	0.01	0.00	0.11	1.15	0.26	0.02	0.00	0.55	0.00	0.03	0.01	1.14	0.83	4.88	1.94	1.41	1.49	3.66
Mollicutes RF39	0.03	0.12	0.11	0.13	0.41	0.65	2.69	4.04	0.72	0.01	0.11	0.17	0.23	0.12	0.17	1.05	1.02	0.88	2.92	0.24	4.36	0.34
Blautia	0.05	0.08	0.02	0.09	0.09	0.05	0.14	0.26	0.17	0.02	0.04	0.01	0.01	0.14	0.01	0.41	0.33	0.18	0.14	0.09	0.06	0.17
Clostridium sensu stricto 1	0.04	0.03	0.03	0.00	0.00	0.00	0.22	0.03	0.03	0.05	0.37	0.01	0.01	0.08	0.00	0.03	0.01	0.06	0.62	0.01	0.72	0.00
Defluviitaleaceae UCG-011	0.02	0.12	0.03	0.10	0.16	0.10	0.17	0.33	0.25	0.04	0.08	0.09	0.19	0.12	0.12	0.68	0.68	0.32	0.02	0.02	0.01	0.00
Enterobacteriaceae	0.02	0.08	0.03	0.00	0.00	0.01	0.01	0.12	0.01	0.02	0.00	0.55	0.00	0.02	0.01	0.10	0.05	0.27	0.13	0.08	0.10	0.14
Erysipelotrichaceae	0.06	0.40	0.15	0.10	0.09	0.02	0.34	0.99	0.23	0.11	0.22	0.21	0.04	0.05	0.12	0.40	0.75	0.54	0.14	0.01	0.14	0.14
Fusicatenibacter	0.02	0.70	0.17	0.12	0.14	0.06	0.30	0.36	0.32	0.07	0.53	0.49	0.12	0.20	0.01	0.50	0.22	0.36	0.21	0.02	0.14	0.01
GCA-900066225	0.01	0.07	0.03	0.05	0.03	0.00	0.05	0.22	0.23	0.06	0.05	0.13	0.03	0.06	0.02	0.20	0.28	0.09	0.02	0.03	0.08	0.00
Parabacteroides	0.00	0.00	0.00	0.04	0.01	0.04	0.00	0.35	1.37	0.00	0.00	0.00	0.00	0.00	0.03	0.00	2.48	1.79	0.00	0.00	0.00	0.00
Phascolarctobacterium	0.00	0.00	0.00	0.00	0.19	0.12	0.00	0.10	0.64	0.00	0.00	0.00	0.00	0.13	0.05	0.00	0.85	0.48	0.00	0.00	0.01	0.00
Ruminococcaceae UCG-013	0.02	0.18	0.04	0.17	0.15	0.05	0.21	0.28	0.26	0.01	0.19	0.11	0.08	0.04	0.14	0.42	0.43	0.30	0.01	0.01	0.05	0.02
Ruminococcus 1	0.10	0.22	0.16	0.01	0.05	0.00	0.33	0.48	0.33	0.07	0.32	0.29	0.01	0.00	0.00	0.53	0.59	0.42	0.00	0.00	0.00	0.00
Shuttleworthia	0.01	0.14	0.05	0.08	0.08	0.05	0.26	0.28	0.28	0.01	0.18	0.08	0.17	0.13	0.03	0.86	0.43	0.21	0.12	0.03	0.02	0.00
Staphylococcus	0.06	0.71	0.03	0.00	0.00	0.00	0.04	0.00	0.00	0.08	2.78	0.03	0.00	0.00	0.00	0.00	0.00	0.19	2.57	0.05	0.47	0.26
Bacillales	0.09	0.17	0.06	0.00	0.00	0.01	0.00	0.02	0.02	0.03	0.11	0.32	0.00	0.00	0.00	0.00	0.02	0.01	0.00	0.02	0.00	0.00

Lactococcus	0.13	0.22	0.52	0.00	0.00	0.00	0.00	0.00	0.00	0.09	0.34	1.84	0.00	0.00	0.00	0.00	0.00	0.00	0.00	0.00	0.00	0.04
Streptococcus	0.34	9.27	0.03	0.00	0.01	0.00	0.01	0.00	0.01	0.10	2.70	0.02	0.00	0.01	0.00	0.00	0.00	0.14	0.09	0.03	0.05	0.02
Clostridium_sensu_stricto_7	0.00	1.81	0.00	0.00	0.00	0.00	0.01	0.00	0.00	0.30	0.00	0.00	0.00	0.00	0.00	0.00	0.01	0.00	0.01	0.00	0.01	0.00
Paraclostridium	0.16	2.65	0.27	0.00	0.00	0.00	0.00	0.00	0.00	0.29	0.20	0.13	0.00	0.00	0.00	0.00	0.01	0.00	0.00	0.00	0.00	0.00
Peptostreptococcaceae	34.98	0.45	0.08	0.00	0.00	0.00	0.01	0.03	0.02	6.28	0.34	0.68	0.01	0.00	0.00	0.03	0.02	0.01	0.00	0.04	0.00	0.01
Romboutsia	3.63	2.63	0.49	0.00	0.00	0.00	0.01	0.00	0.00	8.30	1.46	0.87	0.00	0.00	0.00	0.00	0.00	0.00	0.03	0.00	0.09	0.00
Firmicutes	0.18	0.99	0.16	0.00	0.00	0.00	0.02	0.03	0.05	0.02	0.03	0.46	0.00	0.00	0.00	0.04	0.03	0.03	0.00	0.00	0.00	0.00
Rhodospirillales	0.02	0.38	0.24	0.25	0.08	0.06	0.00	0.00	0.00	0.00	0.47	0.65	0.37	0.04	0.19	0.00	0.00	0.00	0.00	0.00	0.00	0.00

Table S3. For each genus, mean (standard deviation) of relative abundances, limits of the classes after discretization with the R2-GenOpt* algorithm of BaysiaLab and percentages of each class.

Genera	Mean (st. dev.)	Class limits (%)
Alcaligenes	1.12 (4.59)	0 (50.90)
		0-0.11 (29.39)
		>0.11 (19.71)
Alistipes	4.35 (10.60)	0 (48.75)
		0-0.22 (18.64)
		>0.22 (32.62)
Bacteria	0.35 (0.66)	0 (16.49)
		0-0.16 (51.97)
		>0.16 (31.54)
Bacillales	0.04 (0.11)	0 (58.42)
		0-0.05 (22.94)
Bacteroides	2.60 (10.85)	0 (43.37)
		0-0.19 (18.64)
		>0.19 (37.99)
Bacteroidales	0.06 (0.23)	0 (77.78)
		0-0.10 (15.41)
		>0.10 (6.81)
Barnesiella	0.18 (0.73)	0 (82.08)
		0-0.09 (5.73)
		>0.09 (12.19)
Blautia	0.11 (0.17)	0 (26.16)
		0-0.08 (39.07)
		>0.08 (34.77)
Butyricicoccus	0.41 (0.50)	0 (13.26)
		0-0.11 (29.39)

		>0.11 (57.35)
Candidatus Arthromitus	4.40 (10.03)	0 (11.11)
		0-0.25 (32.62)
		>0.25 (56.27)
Candidatus Saccharimonas	0,07 (0,30)	0 (69.18)
		0-0.04 (10.04)
		>0.04 (20.79)
Christensenellaceae R-7 group	0.32 (0.46)	0 (24.01)
		0-0.12 (33.69)
		>0.12 (42.29)
Clostridiales	0.93 (1.40)	0 (35.84)
		0-0.39 (26.16)
		>0.39 (37.99)
Clostridiales_vadinBB60 group	1.25 (1.70)	0 (22.94)
		0-0.33 (27.24)
		>0.33 (49.82)
Clostridium_sensu_stricto_1	0,13 (0,46)	0 (47.67)
		0-0.15 (42.29)
		>0.15 (10.04)
Clostridium_sensu_stricto_7	0.12 (0.68)	0 (89.61)
		0-0.03 (4.66)
		>0.03 (5.73)
Corynebacterium 1	0.11 (0.47)	0 (55.20)
		0-0.12 (35.84)
		>0.12 (0.09)
Defluviitaleaceae_UCG-011	0.14 (0.22)	0 (36.92)
		0-0.08 (20.79)
		>0.08 (42.29)
DTU089	0.31 (0.56)	0 (21.86)
		0-0.08 (22.94)

		>0.08 (55.20)
Eisenbergiella	1.49 (2.31)	0 (8.96)
		0-0.25 (34.77)
		>0.25 (56.27)
Enterobacteriaceae	0.09 (0.26)	0 (34.77)
		0-0.04 (35.84)
		>0.04 (29.39)
Enterococcus	0.33 (1.89)	0 (45.52)
		0-0.13 (35.84)
		>0.13 (18.64)
Erysipelatoclostridium	0.40 (0.72)	0 (5.80)
		0-0.11 (35.14)
		>00.11 (59.06)
Erysipelotrichaceae	0.24 (0.35)	0 (16.49)
		0-0.10 (34.77)
		>0.10 (48.75)
Escherichia-Shigella	1.00 (2.95)	0 (29.39)
		0-0.39 (47.67)
		>0.39 (22.94)
Faecalibacterium	4.39 (7.54)	0 (8.96)
		0-0.27 (31.54)
		>0.27 (59.50)
Firmicutes	0.10 (0.36)	0 (55.20)
		0-0.11 (32.62)
		>0.11 (12.19)
Flavonifractor	0.19 (0.23)	0 (20.79)
		0-0.08 (26.16)
		>0.08 (53.05)
Fournierella	0.07 (0.16)	0 (54.12)
		0-0.09 (29.39)

		>0.09 (16.49)
Fusicatenibacter	0.22 (0.30)	0 (22.94)
		0-0.09 (25.09)
		>0.09 (51.97)
Gastranaerophilales	0.90 (1.92)	0 (33.69)
		0-0.26 (27.24)
		>0.26 (39.07)
GCA-900066225	0.07 (0.11)	0 (44.44)
		0-0.06 (20.79)
		>0.06 (34.77)
GCA-900066575	0.23 (0.29)	0 (24.01)
		0-0.11 (26.16)
		>0.11 (49.82)
Helicobacter	0.15 (0.46)	0 (79.93)
		0-0.16 (8.96)
		>0.16 (11.11)
Lachnospiraceae	9.12 (8.60)	<=1.26 (32.80)
		>1.26 (67.20)
Lactobacillus	37.83 (38.39)	<=4 (38.17)
		>4 (61.83)
Lactococcus	0.14 (0.57)	0 (69.18)
		0-0.06 (11.11)
		>0.06 (19.71)
Megamonas	0.42 (1.86)	0 (85.30)
		0-0.14 (6.81)
		>0.14 (7.89)
Mollicutes_RF39	1.06 (2.98)	0 (19.71)
		0-0.28 (40.14)
		>0.28 (40.14)
Negativibacillus	0.50 (0.70)	0 (20.79)

		0-0.12 (20.79)
		>0.12 (58.42)
Oscillibacter		0 (24.01)
		0-0.13 (17.56)
		>0.13 (58.42)
Parabacteroides	0.28 (1.04)	0 (81.00)
		0-0.27 (8.96)
		>0.27 (10.04)
Paraclostridium	0.20 (1.35)	0 (74.55)
		0-0.14 (15.41)
		>0.14 (10.04)
Peptostreptococcaceae	1.96 (10.52)	0 (47.67)
		0-0.20 (33.69)
		>0.20 (18.64)
Phascolarctobacterium	0.10 (0.29)	0 (78.85)
		0-0.22 (11.11)
		>0.22 (10.04)
Rhodospirillales	0.10 (0.27)	0 (62.72)
		0-0.10 (19.71)
		>0.10 (17.56)
Romboutsia	0.87 (3.24)	0 (61.65)
		0-0.30 (15.41)
		>0.30 (22.94)
Ruminiclostridium_5	0.64 (0.78)	0 (13.26)
		0-0.14 (26.16)
		>0.14 (60.57)
Ruminiclostridium_9	1.03 (1.28)	0 (12.19)
		0-0.17 (27.24)
		>0.17 (60.57)
Ruminococcaceae	6.24 (6.63)	0 (2.51)

		0-0.74 (32.62)
		>0.74 (64.87)
Ruminococcaceae NK4A214 group	0.37 (0.60)	0 (36.92)
		0-0.14 (25.09)
		>0.14 (37.99)
Ruminococcaceae_UCG-005	2.80 (4.53)	0 (16.49)
		0-0.35 (30.47)
		>0.35 (53.05)
Ruminococcaceae_UCG-010	0.15 (0.23)	0 (43.37)
		0-0.13 (22.94)
		>0.13 (33.69)
Ruminococcaceae_UCG-013	0.13 (0.20)	0 (35.84)
		0-0.09 (28.32)
		>0.09 (35.84)
Ruminococcaceae_UCG-014	3.87 (5.17)	0 (5.73)
		0-0.46 (32.62)
		>0.46 (61.65)
Ruminococcus_1	0.17 (0.27)	0 (53.05)
		0-0.09 (16.49)
		>0.09 (30.47)
Sellimonas	0.17 (0.18)	0 (19.71)
		0-0.08 (21.86)
		>0.08 (58.42)
Shuttleworthia		0 (35.84)
		0-0.122 (33.69)
		>0.122 (30.47)
Staphylococcus		0 (58.42)
		0-0.13 (21.86)
		>0.13 (19.71)
Streptococcus	0.64 (3.07)	0 (49.82)

		0- 0.17 (34.77)
		>0.17 (15.41)
Subdoligranulum	1.05 (2.03)	0 (5.73)
		0-0.19 (31.54)
		>0.19 (62.72)
Tyzzerella	0.19 (0.23)	0 (20.79)
		0-0.09 (28.32)
		>0.09 (50.90)

Table S4. Mutual information (MI) and mean relative abundances of the genera in the lowest, middle, highest categories of their latent construct (LC) and the corresponding LC means (in italics). The LC9 has two categories.

**Latent constructs**	**MI**	**Lowest**	**Middle**	**Highest**	**Overall**
LC0		*2.94*	*3.68*	*10.34*	*7 .38*
Candidatus Arthromitus	0.83	0.40	0.07	6 .71	4 .40
Corynebacterium 1	0.74	0.00	0.01	0.03	0.11
Alistipes	0.73	10.48	13.07	0.03	4 .35
Lactobacillus	0.70	1.06	8 .04	57 .07	37 .83
Bacteroides	0.70	6 .22	6 .70	0.29	2.60
Enterococcus	0.50	0.01	0.03	0.36	0.33
Bacteroidales	0.14	0.02	0.46	0.01	0.06
LC1		*0.02*	*0.30*	*1.99*	*0.51*
Firmicutes	0.81	0.00	0.00	0.43	0.10
Peptostreptococcaceae	0.81	0.03	0.61	8 .14	1.96
Staphylococcus	0.74	0.00	0.93	1.19	0.37
Ruminococcus 1	0.74	0.01	0.00	0.24	0.17
Romboutsia	0.73	0.14	0.04	3.47	0.87
Lactococcus	0.63	0.03	0.01	0.72	0.14
Streptococcus	0.53	0.02	0.93	2.62	0.64
Bacillales	0.53	0.00	0.02	0.17	0.04
Paraclostridium	0.53	0.00	0.01	0.71	0.20
**LC2**		*0.01*	*0.08*	*0.94*	*0.58*
Clostridiales vadinBB60 group	1.09	0.01	0.13	2.38	1.25
Flavonifractor	0.94	0.00	0.04	0.31	0.19
Ruminiclostridium 5	0.91	0.02	0.20	1.04	0.64
Butyricicoccus	0.90	0.03	0.04	0.66	0.41
Sellimonas	0.89	0.01	0.07	0.28	0.17
Ruminococcaceae UCG-013	0.84	0.01	0.05	0.25	0.13
Mollicutes RF39	0.59	0.07	0.07	1.56	1.06

LC3		*0.27*	*0.34*	*4.51*	*3.24*
Oscillibacter	0.84	0.01	0.04	0.51	0.34
Ruminiclostridium 9	0.80	0.03	0.05	1.57	1.03
Ruminococcaceae	0.78	0.76	0.29	9 .32	6 .24
Lachnospiraceae	0.76	1.21	1.44	13.34	9 .12
DTU089	0.73	0.00	0.07	0.46	0.31
Subdoligranulum	0.66	0.06	0.30	1.53	1.05
LC4		*0.13*	*0.85*	*3.16*	*1.34*
Escherichia-Shigella	1.00	0.11	3.44	4 .10	1.01
Ruminococcaceae UCG-005	0.97	0.61	0.52	5 .18	2.80
Faecalibacterium	0.88	0.12	0.11	7 .28	4 .39
Enterobacteriaceae	0.80	0.02	0.28	0.23	0.09
Gastranaerophilales	0.77	0.04	0.05	1.23	0.90
Rhodospirillales	0.75	0.00	0.01	0.00	0.10
Candidatus Saccharimonas	0.71	0.00	0.01	0.00	0.07
LC5		*0.02*	*1.01*	*1.16*	*0.32*
Phascolarctobacterium	1.00	0.00	0.07	0.80	0.10
Helicobacter	0.80	0.02	0.77	0.01	0.15
Barnesiella	0.76	0.00	0.99	0.02	0.18
Parabacteroides	0.67	0.08	0.06	1.85	0.28
Megamonas	0.52	0.01	2.29	0.06	0.42
**LC6**		*0.06*	*0.23*	*0.58*	*0.32*
Ruminococcaceae NK4A214 group	1.00	0.05	0.71	0.06	0.37
Ruminococcaceae UCG-010	1.00	0.05	0.06	0.05	0.15
Christensenellaceae R-7 group	0.70	0.19	0.55	0.03	0.32
Fournierella	0.44	0.00	0.21	0.00	0.07
Alcaligenes	0.36	0.03	0.07	2.81	1.12
**LC7**		*0.02*	*0.06*	*0.44*	*0.23*
Shuttleworthia	1.00	0.00	0.02	0.39	0.14
Fusicatenibacter	0.83	0.00	0.03	0.40	0.22

GCA-900066575	0.79	0.01	0.05	0.40	0.23
Negativibacillus	0.73	0.07	0.14	0.83	0.50
Tyzzerella	0.70	0.03	0.07	0.34	0.19
Erysipelotrichaceae	0.55	0.02	0.04	0.42	0.24
**LC8**		0.02	0.20	0.76	0.38
Defluviitaleaceae UCG-011	1.00	0.00	0.07	0.28	0.14
Clostridiales	0.68	0.03	0.47	1.89	0.93
GCA-900066225	0.56	0.01	0.02	0.15	0.07
Bacteria	0.44	0.03	0.20	0.67	0.35
**LC9**		*0.06*		*0.44*	*0.27*
Erysipelatoclostridium	1.00	0.09		0.64	0.41
Blautia	0.81	0.01		0.19	0.11
**LC10**		*0.06*	*0.12*	*4 .14*	*2.43*
Eisenbergiella	1.00	0.03	0.08	2.59	1.49
Ruminococcaceae UCG-014	0.86	0.11	0.14	6 .19	3.87
